# A phase II study of cisplatin, vindesine and continuously infused 5-fluorouracil in the treatment of advanced non-small-cell lung cancer. Osaka Lung Cancer Chemotherapy Study Group.

**DOI:** 10.1038/bjc.1996.211

**Published:** 1996-05

**Authors:** T. Nakano, H. Ikegami, S. Nakamura, T. Kawase, H. Nishikawa, S. Yokota, M. Yoshida, T. Tachibana, T. Igarashi, K. Komuta, K. Higashino

**Affiliations:** Third Department of Internal Medicine, Hyogo College of Medicine, Japan.

## Abstract

Fifty-two previously untreated patients with advanced non-small-cell lung cancer (NSCLC) were treated on a 14 day cycle with cisplatin (60 mg m-2 i.v.) and vindesine (3 mg m-2 i.v.) on day 1, followed by a 3 day continuous infusion of 5-fluorouracil (800 mg m-2 day-1) starting on day 8. An overall response rate of 40.4% was observed in 47 evaluable patients, which included one complete response and 18 partial responses. Responses were achieved in 61.1% of stage 3 patients and 27.6% of stage 4 patients. The median progression-free interval was 19.3 weeks, and median survival time was 41.6 weeks (47.1 weeks for patients with stage 3 disease and 38.7 weeks for those with stage 4 disease). Toxicity was well tolerated. Gastrointestinal and renal toxicities did not exceed WHO grade 2. Grade 3 or 4 leucopenia and anaemia occurred in nine (19%) and four (9%) patients respectively, but only grade 2 thrombocytopenia was observed. Phlebitis at the infusion site was observed in 24 patients (53%). This treatment programme achieved a response rate similar to other active combination regimens for the treatment of advanced NSCLC, and was less toxic.


British Journal of Cancer (1996) 73, 1096-1100
© 1996 Stockton Press All rights reserved 0007-0920/96 $12.00

### A phase II study of cisplatin, vindesine and continuously infused 5-fluorouracil in the treatment of advanced non-small-cell lung cancer

Osaka Lung Cancer Chemotherapy Study Group. T Nakano[1], H Ikegami[2], S Nakamura[2], I Kawase[3], H Nishikawa[4], S Yokota[4], M Yoshida[5], T Tachibana[5], T Igarashi[6], K Komuta[6] and K Higashino[1]

*[1]Third Department of Internal Medicine, Hyogo College of Medicine, Hyogo; [2]Fourth Department of Internal Medicine, Center for Adult Disease, Osaka; [3]Third Department of Internal Medicine, Osaka University Medical School, Osaka; [4]Department of Internal Medicine, Toneyama National Hospital, Osaka; [5]Department of Internal Medicine, Osaka Prefectural Hospital, Osaka; [6]Second Department of Internal Medicine, Osaka Teishin Hospital, Osaka, Japan.*

**Summary** Fifty-two previously untreated patients with advanced non-small-cell lung cancer (NSCLC) were treated on a 14 day cycle with cisplatin (60 mg $m^{-2}$ i.v.) and vindesine (3 mg $m^{-2}$ i.v.) on day 1, followed by a 3 day continuous infusion of 5-fluorouracil (800 mg $m^{-2}$ $day^{-1}$) starting on day 8. An overall response rate of 40.4% was observed in 47 evaluable patients, which included one complete response and 18 partial responses. Responses were achieved in 61.1% of stage 3 patients and 27.6% of stage 4 patients. The median progression-free interval was 19.3 weeks, and median survival time was 41.6 weeks (47.1 weeks for patients with stage 3 disease and 38.7 weeks for those with stage 4 disease). Toxicity was well tolerated. Gastrointestinal and renal toxicities did not exceed WHO grade 2. Grade 3 or 4 leucopenia and anaemia occurred in nine (19%) and four (9%) patients respectively, but only grade 2 thrombocytopenia was observed. Phlebitis at the infusion site was observed in 24 patients (53%). This treatment programme achieved a response rate similar to other active combination regimens for the treatment of advanced NSCLC, and was less toxic.

**Keywords:** non-small-cell lung cancer; combination chemotherapy; cisplatin; vindesine; continuous infusion; 5-fluorouracil

The use of drug combinations in the treatment of advanced non-small-cell lung cancer (NSCLC) has been intensively investigated. Their use, however, remains controversial. Cisplatin (CDDP) in combination with vinca alkaloids, vindesine (VDS) or vinblastine (VBL), has been widely used for induction treatment of NSCLC since Gralla *et al.* (1981) first demonstrated a response rate of 43%. Response rates of approximately 30% have since been shown in advanced NSCLC using a combination of VDS + CDDP (VP) (Elliott *et al.*, 1984; Dhingra *et al.*, 1985; Kawahara *et al.*, 1991). Treatment regimens in which one further active chemotherapeutic agent has been added to vinca alkaloid and cisplatin have been tried in an attempt to improve response and survival rates. The combination of mitomycin with VP (MVP) has been reported to achieve response rates of 20-61% (Kris *et al.*, 1986; Einhorn *et al.*, 1986; Miller *et al.*, 1986; Joss *et al.*, 1990; Fukuoka *et al.*, 1991). Although these intensive combination chemotherapy regimens achieve an improvement in objective response, serious drug-related toxicity is often experienced. Active chemotherapeutic regimens that are less toxic would therefore be desirable.

Although 5-fluorouracil (5-FU) shows limited activity as a single agent in NSCLC, it reacts synergistically with CDDP against murine tumours (Schabel *et al.*, 1979; Mabel and Little, 1979). This has prompted clinical studies of the combination of CDDP and 5-FU against NSCLC. Weiden *et al.* (1985) have reported a response rate of 37% in NSCLC using this combination. Continuous infusion (CI) of 5-FU has been found to increase the therapeutic response and decrease myelosuppression compared with the use of bolus injections (Seifert *et al.*, 1975; Kish *et al.*, 1985). Decker *et al.* (1983) found the combination of CDDP and a 5 day infusion of 5-FU useful in the treatment of head and neck cancers. They reported a 94% response rate, including a complete response rate of 63%. In a pilot study by the Mid-Atlantic Oncology Program, CDDP (120 mg $m^{-2}$) infused over 24 h + CI 5-FU gave a response rate of 47% in advanced NSCLC (Heim *et al.*, 1986). Toxicology profiles of these CDDP + 5-FU regimens indicate less haematological and nephrological toxicity than standard dose VP and MVP regimens. However, a higher incidence of severe mucositis was observed, and, if doxorubicin was also included, clinically significant myelosuppression, similar to that encountered with VP and MVP regimens, was also experienced (Ruckdeschel *et al.*, 1981). Although improved response rates have been achieved, an optimum dose and schedule has yet to be identified.

These reports encouraged us to perform a phase II trial of CDDP and VDS in combination with CI 5-FU against advanced NSCLC. We decided to administer VP separately from CI 5-FU, in an attempt to minimise toxicity without affecting efficacy. In most of the earlier studies CDDP and 5-FU were administered on the same day. The trial was designed so that CDDP + VDS and CI 5-FU were injected on alternate weeks. The immediate objectives were to determine the major objective response rate to this treatment programme, and to define the toxicities involved. The activity of this combination against advanced NSCLC has not been studied previously.

### Patients and methods

### *Patient eligibility*

Our criteria for eligibility were; cytologically or histologically confirmed NSCLC; measurable disease; and Eastern Co-operative Oncology Group (ECOG) performance status (PS) score of less than 3; age less than 75 years; no evidence of CNS metastases; adequate renal (serum creatinine < 1.5 mg $dl^{-1}$, BUN < 25 mg $dl^{-1}$), hepatic (serum bilirubin < 1.5 mg $dl^{-1}$, sGOT and sGPT values less than twice the norm for the institution) and bone marrow function (WBC > 4000 $mm^{-3}$, platelets > 100 000 $mm^{-3}$; no prior chemotherapy; absence of other concurrent active malignancies; absence of superior vena cava syndrome; accessibility for follow-up; and informed consent to participate in this study.

Correspondence: T Nakano, Third Department of Internal Medicine, Hyogo College of Medicine, 1-1 Mukogawa-cho, Nishinomiya, Hyogo 663, Japan
Received 7 October 1994; revised 23 June 1995; accepted 29 November 1995

### *Pretreatment and follow-up studies*

Pretreatment evaluation involved taking the patient history, physical examination, complete blood cell counts (CBCs), a blood chemistry profile, an electrocardiogram (ECG), complete urinalysis, bone marrow examination, bone scintigraphy and chest radiograph and computerised tomography of the chest and brain, as well as an ultrasonic study of the abdomen. A clinical assessment and a plain chest radiograph were performed each week. In addition, CBC and biochemical tests were performed twice a week.

Patients were staged according to the TNM classification system (Mountain, 1986). The severity of drug toxicity was graded according to the WHO toxicity scales (Miller *et al.*, 1981).

### *Drug administration*

The treatment scheme is summarised in Figure 1.

The chemotherapy was given intravenously as follows: CDDP (60 mg $m^{-2}$) in 500 ml of normal saline over 1 h + VDS (3 mg $m^{-2}$) by bolus injection on day 1; and 5-FU (800 mg $m^{-2}$ $day^{-1}$) by continuous 3 day infusion into a peripheral vein starting on day 8 (PVF). This treatment was repeated every 14 days for at least three cycles, so that CDDP and VDS alternated weekly with a 3 day continuous infusion of 5-FU. A 25% reduction in drug dosage was required in subsequent cycles for those patients who experienced WHO grade 4 myelotoxicity, grade 2 nephrotoxicity, and/or another severe treatment-related toxicity. For patients who achieved a response, the treatment was continued for an additional three cycles. All patients received at least 2.5 l of hydration on the day of CDDP administration with forced diuresis. Treatment then continued until tumour progression was observed, or until no further response was achieved over three successive cycles.

Concomitant radiation therapy to the brain and bone was permitted. Granulocyte colony-stimulating factor (G-CSF) and other cytokines were not used in this study. Antiemetic therapy, using methylprednisolone and metoclopramide, was given at the discretion of the investigator.

### *Response criteria*

Patients were evaluated for response after three treatment cycles. A complete response (CR) was defined as complete disappearance of all measurable disease for at least 4 weeks without appearance of any new lesions. A partial response (PR) was defined as a 50% or more reduction in the sum of the products of the two longest perpendicular diameters of measurable lesions for a least 4 weeks; no change (NC) was defined as a reduction of less than 50% to an increase of less than 25% in measurable disease, without the appearance of new lesions. Progressive disease (PD) was defined as an increase of more than 25% in the size of any measurable lesion, or the appearance of new lesions.

Survival was calculated from the start of treatment to death or the date of the last follow-up, using the actuarial method of Kaplan-Meier. The exact confidence intervals for the response rates were calculated according to the formula proposed by Ghosh (1979).

### Results

### *Patient characteristics*

Fifty-two patients were entered into this study between February 1986 and October 1992.

Patient characteristics are summarised in Table I. Of the 52 patients registered, five were ineligible: two had received prior chemotherapy; two were more than 75 years old; and one had another concomitant malignant neoplasm. Of the 47 evaluable patients, 83% (39/47) were men. A total of 53% of cases (25/47) were adenocarcinoma, 34% (16/47) were squamous cell carcinoma and 13% (6/47) were large-cell carcinoma. A total of 17% of patients (8/47) had clinical stage 3A disease, 21% (10/47) has stage 3B disease and 62% (29/47) had stage 4 disease. A total of 79% (37/47) had a PS score of 0-1. The median number of treatment cycles administered was three (range 1-6). Two out of the 18 patients with stage 3 disease received a full dose of thoracic irradiation (5000 cGy) after completing the chemotherapy.

### *Response*

The objective responses are shown in Table II. Of the 47 evaluable patients, one patient achieved CR (2%) and 18 patients achieved PR (38.3%), giving an overall response rate of 40.4% (95% confidence interval, 26.3-54.5%). NC was documented in 23 cases (48.9%), and PD in three cases (6.4%). Two other patients died of other diseases (one patient died of cerebral haemorrhage and the other died of diabetic complications). Objective responses were observed in 9/16 cases of squamous cell carcinoma (56.3%) and in 10/31 cases of non-squamous cell carcinoma (32.3%). The response rates for patients with stage 3 and stage 4 disease were 61.1% and 27.6% respectively. For stage 4 disease, 42.9% of squamous cell carcinoma patients achieved remissions, almost twice the figure for non-squamous cell carcinoma patients (22.7%). For patients with stage 3 disease, the response rates were 66.7% and 55.5% respectively. The median progression-free interval was 19.3 weeks.

Thirty-three out of 47 patients had died by the time of analysis. The overall median survival for the 47 patients was 41.6 weeks. The median survival for patients with stage 3

**Figure 1** Schema of the treatment protocol.

**Table I** Patient characteristics

| *Characteristics* | *No. of patients* |
|---|---|
| Patients | |
| Entered/evaluable | 52/47 |
| Male/female | 39/8 |
| Age | |
| Median (range) | 65 (48-74) |
| Histology | |
| Adenocarcinoma | 25 |
| Squamous cell carcinoma | 16 |
| Large cell carcinoma | 6 |
| Performance status (ECOG) | |
| 0-1 | 37 |
| 2 | 9 |
| 3 | 1 |
| Clinical stage | |
| III A | 8 |
| III B | 10 |
| IV | 29 |

Table II Tumour response and survival of 47 evaluable patients

| Stage/ histology | Number of patients | Response CR | PR | Response rate (%) | Survival 1 year (%) | 2 year (%) |
|---|---|---|---|---|---|---|
| Stage | | | | | | |
| III | 18 | 1 | 10 | 61.1 | 65.4 | 35.0 |
| IV | 29 | | 8 | 27.6 | 40.0 | 10.8 |
| Histology | | | | | | |
| Squamous cell carcinoma | | | | | | |
| III | 9 | 1 | 5 | 66.7 | 62.2 | 20.7 |
| IV | 7 | | 3 | 42.9 | 57.1 | 19.1 |
| III+IV | 16 | 1 | 8 | 56.3 | 60.0 | 14.3 |
| Adenocarcinoma | | | | | | |
| III | 8 | | 5 | 62.5 | 83.3 | 62.5 |
| IV | 17 | | 5 | 29.4 | 28.6 | 14.3 |
| III+IV | 25 | | 10 | 40.0 | 43.8 | 26.3 |
| Large cell carcinoma | | | | | | |
| III | 1 | | | 0 | 0 | 0 |
| IV | 5 | | | 0 | 50.0 | 0 |
| III+IV | 6 | | | 0 | 40.0 | 0 |
| Non-Sq | | | | | | |
| III | 9 | | 5 | 55.5 | 71.4 | 53.6 |
| IV | 22 | | 5 | 22.7 | 33.4 | 7.4 |
| III+IV | 31 | | 10 | 32.3 | 43.5 | 17.9 |
| Total | 47 | 1 | 18 | 40.4 (95% Confidence interval 26.3-54.5) | 49.0 | 17.0 |

CR, complete response; PR, partial response; Non-sq, adenocarcinoma + large cell carcinoma.

Table III Toxicity ($n=47$)

| Toxicity | WHO grade 1 | 2 | 3 | 4 |
|---|---|---|---|---|
| Leucopenia | 9 (19) | 18 (38) | 8 (17) | 1 (2) |
| Thrombocytopenia | 7 (15) | 2 (4) | 0 | 0 |
| Anaemia (Hb) | 26 (55) | 9 (19) | 4 (9) | 0 |
| Nephrotoxicity (creatinine) | 2 (4) | 0 | 0 | 0 |
| Hepatotoxicity (sGOT/GPT) | 9 (19) | 1 (2) | 0 | 0 |
| Vomiting/nausea | 14 (30) | 7 (15) | 0 | 0 |
| Diarrhoea | 7 (15) | 0 | 0 | 0 |
| Alopecia | 16 (34) | 12 (26) | 2 (4) | 0 |
| Peripheral neuropathy | 8 (17) | 1 (2) | 0 | 0 |
| Fever | 11 (23) | 4 (9) | 0 | 0 |
| Phlebitis[a] | 13 (28) | 11 (23) | | |

Values represent number of patients (%). [a]Phlebitis: grade 1, mild; grade 2, moderate.

disease was 47.1 weeks, compared with 38.7 weeks for those with stage 4 disease. No significant difference in survival was observed between the patients with squamous cell carcinoma (median survival 50.6 weeks) and those with non-squamous cell carcinoma (median survival 38.9 weeks). There were too few patients to allow us to attach statistical significance to the difference in survival by histology. However, the 1 year and 2 year survival rates for patients with stage 3 squamous cell carcinoma were 62.2% and 20.7% respectively, and 71.4% and 53.6% respectively for patients with stage 3 non-squamous cell carcinoma. For patients with stage 4 disease, the 1 year and 2 year survival rates were respectively 57.1% and 19.1% for squamous cell carcinoma, and 33.4% and 7.4% for non-squamous cell carcinoma.

*Toxicity*

The toxicities encountered are summarised in Table III. The most frequent major toxic effect was myelosuppression, but this was of a mild degree. Grade 3 or 4 leucopenia was observed in nine patients (19%), but only grade 2 thrombocytopenia was observed and only in two patients (4%). Grade 1 or 2 anaemia occurred in 35 patients (74%) and grade 3 or 4 in four patients (9%). Non-haematological toxicity was generally mild: 21 patients (45%) suffered vomiting, but this never exceeded grade 2; grade 1 diarrhoea was observed in seven patients (15%); and peripheral neuropathy in nine patients (19%). A transient rise in serum creatinine levels (>2.0 mg $ml^{-1}$) occurred in two patients (4%), and elevation of sGOT and/or sGPT (>100 U $L^{-1}$) in one patient (2%), none of whom required specific therapy. No stomatitis was observed. Phlebitis at the site of drug administration was noted in 24 patients (51%); all of these improved under conservative management. Pigmentation of the overlying skin was seen in 18 (38%) patients. Grade 1 alopecia was seen in 16 patients (34%) and grade 2 in 12 patients (26%).

### Discussion

The observed response rate of 40.4% with a median survival of 41.6 weeks for patients with advanced NSCLC receiving PVF therapy is similar to that recently reported with several investigators (Fukuoka *et al.*, 1991, 1992) for the MVP regimen using CDDP in moderate doses (80 mg $m^{-2}$). Patients with clinical stage 3 and stage 4 disease were included in this trial. Objective responses were achieved in 11/18 (61.1%) stage 3 patients and in 8/29 (27.6%) stage 4 patients. In the report on the MVP regimen using a moderate CDDP dose, the response rates for stage 3 and stage 4 NSCLC were 44% and 56% respectively (Fukuoka *et al.*, 1992).

The combination of CDDP + 5-FU has been most effective against squamous cell lung cancer (Weiden *et al.*, 1985), and squamous cell head and neck cancer (Decker *et al.*, 1983). Klastersky *et al.* (1990) have demonstrated that squamous cell carcinoma is associated with a significantly higher response rate than adenocarcinoma in a randomised study comparing CDDP or carboplatin with etoposide in patients with NSCLC. We achieved a better response rate in squamous cell carcinoma (56.3%) than in non-squamous cell carcinoma (32.3%), using our PVF therapy.

Fukuoka *et al.* (1992) reviewed the results of MVP therapy for advanced NSCLC, and showed that MVP regimens using moderate- or high-dose CDDP (75–120 mg $m^{-2}$) achieved a response rate of 49%, whereas the rate was 30% for low-dose CDDP (60 mg $m^{-2}$ or less). They concluded that CDDP should be given at a dose of 75 mg $m^{-2}$ or more. To maximise chemotherapeutic activity against NSCLC, many investigators are seeking maximal dose intensity for the drug combinations. However, this strategy is often associated with severe toxicity. Weekly administration and an intravenous CI schedule for several agents may improve efficacy and decrease toxicity, compared with the use of conventional i.v. bolus injections (Jacobs *et al.*, 1978; Carlson and Sikic, 1983; Vogelzang, 1984; Saito *et al.*, 1990; Miles *et al.*, 1991). For some drugs, conventional intermittent scheduling every 3–4 weeks may be inferior to alternative means of drug delivery (O'Dwyer and Comis, 1989). In a randomised trial comparing CDDP + CI 5-FU with CDDP + bolus injections of 5-FU, the latter treatment schedule was clearly inferior in terms of the response achieved (Kish *et al.*, 1985). The major advantage of using a CI delivery of 5-FU instead of bolus injection is a marked reduction in bone marrow toxicity (Sikic, 1986). In 1991, the Cancer and Leukaemia Group B reported the result of a random study comparing CDDP + CI 5-FU with CDDP + CI 5-FU + VBL for NSCLC, which showed that neither regimen was effective, and that toxicities were more frequent and more severe in the latter (Richards *et al.*, 1991). However, their treatment was administered using a standard intermittent schedule every 4 weeks.

To minimise toxicity, we separated the administration of CDDP + VDS and CI 5-FU in the treatment schedule, so that CDDP + VDS and a 3 day CI 5-FU were given on alternate weeks. Although this PVF schedule employed a low dose of CDDP (60 mg $m^{-2}$), the response rate was better than that to CDDP (100 mg $m^{-2}$) + CI 5-FU (Weiden *et al.*, 1985), or to a 24 h infusion of CDDP (100 mg $m^{-2}$) + CI 5-FU + etoposide (Rosenthal *et al.*, 1992). Furthermore, our results were similar to those observed using a weekly schedule of CDDP and CI 5-FU + VBL, and to those using CI CDDP + 5-FU + bolus methotrexate (Lynch *et al.*, 1992).

The toxicity profile for the weekly CDDP-based regimen used in the Southwest Oncology Group Study was similar to the standard-dose regimen (Higano *et al.*, 1991). The most active VP and MVP regimens for advanced NSCLC also cause moderate or severe myelosuppression (Luedke *et al.*, 1990; Fukuoka *et al.*, 1992). However, the incidence of severe haematotoxicity was low and well tolerated using our PVF regimen. Vomiting was experienced by most patients, but never exceeded grade 2 and could easily be controlled with antiemetics. The development of chemical phlebitis at the infusion site occurred in 51% of the patients, which sometimes necessitated the replacement of the peripheral venous catheter. Phlebitis has been reported in 45% of patients treated with CDDP + CI 5-FU (Verweij *et al.*, 1989). A high risk of cardiovascular toxicity has also been reported for 5-FU (Pottage *et al.*, 1978; Labianca *et al.*, 1982), but we observed no such side-effect.

There were two deaths from other diseases during our study. One hypertensive patient, whose WBC and thrombocyte count were normal, died of a cerebral haemorrhage. The other died of a diabetic complication, but without any indication of the haematological or non-haematological events associated with the drugs administered. These deaths cannot therefore be attributed to drug-related toxicity.

In conclusion, this PVF regimen for the treatment of advanced NSCLC achieved a response rate similar to other active combination regimens, and was less toxic.

**Acknowledgements**
We are very grateful to Shinsuke Tamura, MD for his many constructive suggestions. The following investigators participated in this study: F Imamura, M Nishio, T Kumagai, M Okuda, S Hosoe, Y Shigedo, S Saito, T Ohzaki, M Takenaka, T Fujisawa, N Okuda, T Ohkawa, A Hayashi, T Koh, S Iwasaki, M Nato, T Hidaka, T Iwasa, M Mikami, T Tsutsui, A Tonomura, Y Hyodo, T Nishian, K Ninomiya and H Fujioka.

### References

CARLSON RW AND SIKIC BI. (1983). Continuous infusion or bolus injection in cancer chemotherapy. *Ann. Intern. Med.*, **99**, 823–833.

DECKER DA, DRELICHMAN A, JACOBS J, HOSCHNER J, KINZIE J, LOH JJ, WEAVER A AND AL-SARRAF M. (1983). Adjuvant chemotherapy with cis-diammino-dichloroplatinum II and 120-hour infusion 5-fluorouracil in stage III and IV squamous cell carcinoma of the head and neck. *Cancer*, **51**, 1353–1355.

DHINGRA HM, VALDIVIESO M, CARR DT, CHIUTEN DF, FARHA P, MURPHY WK, SPITZER G AND UMSAWASDI T. (1985). Randomized trial of three combinations of cisplatin with vindesine and/or VP-16-213 in the treatment of advanced non-small cell lung cancer. *J. Clin. Oncol.*, **3**, 176–183.

EINHORN LH, LOEHRER PJ, WILLIAMS SD, MEYERS S, GABRYS T, NATTAN SR, WOODBURN T, DRASGA R, SONGER J, FISHER W, STEPHENS D AND HUI S. (1986). Random prospective study of vindesine versus vindesine plus high-dose cisplatin versus vindesine plus cisplatin mitomycin C in advanced non-small-cell lung cancer. *J. Clin. Oncol.*, **4**, 1037–1043.

ELLIOTT JA, AHMEDZAI S, HOLE D, DORWARD AJ, STEVENSON RD, KAYS SB, BANHAM SW, STACK BHR AND CALMAN KC. (1984). Vindesine and cisplatin combination chemotherapy compared with vindesine as a single agent in the management of non-small cell lung cancer: a randomized study. *Eur. J. Cancer. Clin. Oncol.*, **8**, 1025–1032.

FUKUOKA M, MASUDA N, FURUSE K, NEGORO S, TAKADA M, MATSUI K, TAKIFUJI N, KUDOH S, KAWAHARA M, OGAWARA M, KODAMA N, KUBOTA K, YAMAMOTO M AND KUSUNOKI Y. (1991). A randomized trial inoperable non-small cell lung cancer. Vindesine and cisplatin versus mitomycin, vindesine and cisplatin versus etoposide and cisplatin alternating with vindesine and mitomycin. *J. Clin. Oncol.*, **9**, 606–613.

FUKUOKA M, NEGORO S, MASUDA N, KUSUNOKI Y, MATSUI K, RYU S, TAKIFUJI N, KUDOH S AND TAKADA M. (1992). Mitomycin C, vindesine, and cisplatin in advanced non-small cell lung cancer. A phase II study. *Am. J. Clin. Oncol.*, **15**, 18–22.

GHOSH BK. (1979). A comparison of some approximate confidence intervals for binomial parameter. *J. Am. Stat. Assoc.*, **74**, 894–900.

GRALLA RJ, CASPER ES, KELSEN DP, BRAUN DW, DUKEMAN ME, MARTINI N, YOUNG CW AND GOLBEY RB. (1981). Cisplatin and vindesine combination chemotherapy for advanced carcinoma of the lung: a randomized trial investigating two dosage schedules. *Ann. Intern. Med.*, **95**, 414–420.

HEIM W, BRERETON HD AND SHEBAUGH D. (1986). Infusional high dose cisplatinum and 5-fluorouracil in advanced non-small cell lung cancer: a Mid-Atlantic Oncology Program pilot study (abstr). *Proc. Am. Soc. Clin. Oncol.*, **5**, 175.

HIGANO CS, CROWLEY J, LIVINGSTON RB, GOODWIN JW, BARLOGIE B AND STUCKEY WJ. (1991). A weekly cisplatin-based induction regimen for extensive non-small cell lung cancer. A Southwest Oncology Group Study. *Cancer*, **67**, 2439–2442.

JACOBS C, BERTINO JR, GOFFINET DR, FEE WE AND GOODE RL. (1978). 24-hours infusion of cis-platinum in head and neck cancers. *Cancer*, **42**, 2135–2140.

JOSS RA, BURKI K, DALQUEN P, SCHATZMANN E, LEYVRAZ S, CAVALLI F, LUDWIG C, SIEGENTHALER P, ALBERTO P, STAHEL R, HOLDENER EE AND SENN H. (1990). Combination chemotherapy with mitomycin, vindesine and cisplatin for non-small cell lung cancer. Association of antitumour activity with initial tumour burden and treatment center. *Cancer*, **65**, 2426–2434.

KAWAHARA M, FURUSE K, KODAMA N, YAMAMOTO M, KUBOTA K, TAKADA M, NEGORO S, KUSUNOKI Y, MATUI K, TAKIFUJI N AND FUKUOKA M. (1991). A randomized study of cisplatin versus cisplatin plus vindesine for non-small lung carcinoma. *Cancer*, **68**, 714–719.

KISH JA, ENSLEY JF, JACOBS J, WEAVER A, CUMMINGS G AND AL-SARRAF M. (1985). A randomized trial of cisplatin (CACP) + 5-fluorouracil (5-FU) infusion and CACP + 5-FU bolus for recurrent and advanced squamous cell carcinoma of the head and neck. *Cancer*, **56**, 2740–2744.

KLASTERSKY J, SCULIER JP, LACROIX H, DABOUIS G, BUREAU G, LIBERT P, RICHEZ M, RAVEZ P, VANDERMOTEN G, THIRIAUX J, CORDIER R, FINET C, BERCHIER MC, SERGYSELS R, MOMMEN P AND PAESMANS M. (1990). A randomized study comparing cisplatin or carboplatin with etoposide in patients with advanced non-small lung cancer: European organization for research and treatment of cancer protocol 07861. *J. Clin. Oncol.*, **8**, 1556–1562.

KRIS MG, GRALLA RJ, WERTHEIM MS, KELSEN DP, O'CONNELL JP, BURKE MT, FIORE JJ, CIBAS IR AND HEELAN RT. (1986). Trial of the combination of mitomycin, vindesine and cisplatin in patients with advanced non-small cell lung cancer. *Cancer Treat. Rep.*, **70**, 1091–1096.

LABIANCA R, BERETTA G, CLERICI M, FRASCHINI P AND LUPORINI G. (1982). Cardiac toxicity of 5-fluorouracil: a study on 1083 patients. *Tumori*, **68**, 505–510.

LUEDKE DW, EINHORN L, OMURA GA, SARMA PR, BARTOLUCCI AA, BIRCH R AND GRECO FA. (1990). Randomised comparison of two combination regimens versus minimal chemotherapy in non-small-cell lung cancer: A Southeastern Cancer Study Group trial. *J. Clin. Oncol.*, **8**, 886–891.

LYNCH TJ, CLARK JR, KALISH LA, FALLON BG, ELIAS AD, SKARIN A AND FREI E. (1992). Continuous-infusion cisplatin, 5-fluorouracil, and bolus methotrexate in the treatment of advanced non-small cell lung cancer. *Cancer*, **70**, 1880–1885.

MABEL JA AND LITTLE AD. (1979). Therapeutic synergism in murine tumors for combination of cis-Diaminechloroplatinum with VP-16 or BCNU (abstr). *Proc. Am. Assoc. Cancer Res.*, **20**, 230.

MILES DW, EARL HM, SOUHAMI RL, HARPER PG, RUDD R, ASH CM, JAMES L, TRASK CWL, TOBIAS JS AND SPIRO SG. (1991). Intensive weekly chemotherapy for good prognosis patients with small-cell lung cancer. *J. Clin. Oncol.*, **9**, 280–285.

MILLER AB, HOOGSTRATEN B, STAQUET M AND WINKLER A. (1981). Reporting results of cancer treatment. *Cancer*, **47**, 207–214.

MILLER TP, VANCE RB, AHMANN FR AND RODNEY SR. (1986). Extensive non-small cell lung cancer treated with mitomycin, cisplatin, and vindesine (MiPE): A Southwest Oncology Group study. *Cancer Treat. Rep.*, **70**, 1101–1104.

MOUNTAIN CF. (1986). A new international staging system for lung cancer. *Chest*, **89**, 225S–233S.

O'DWYER PJ AND COMIS RL. (1989): Schedule as a determinant of cytotoxic drug activity. *Curr. Opin. Oncol.*, **1**, 174–178.

POTTAGE A, HOLT S, LUDGATE S AND LANGLANDS AO. (1978). Fluorouracil cardiotoxicity. *Br. Med. J.*, **1**, 547.

RICHARDS F, PERRY DJ, GOUTSOU M, MODEAS C, MUCHMORE E, REGE V, CHAHINIAN AP, HIRSH V, POIESZ B AND GREEN MR. (1991). Chemotherapy with 5-fluorouracil (5-FU) and cisplatin or 5-FU, cisplatin, and vinblastine for advanced non-small cell lung cancer. A randomized phase II study of the Cancer and Leukemia Group B. *Cancer*, **67**, 2974–2979.

ROSENTHAL CJ, WAMPLER GL, BRERETON HD, AHLGREN JD, LOKICH JJ, FRYER JG AND ALT DE. (1992). A study of oral etoposide, infusional cisplatin, and infusional 5-fluorouracil for locally advanced or metastatic non-small-cell lung cancer. A Mid-Atlantic Oncology Program Study. *Am. J. Clin. Oncol.*, **15**, 12–17.

RUCKDESCHEL JC, MEHTA CR, SALAZAR OM, COHEN M, VOGL S, KOONS LS AND LERNER H. (1981). Chemotherapy for inoperable, non-small cell bronchogenic carcinoma: EST 2575, Generation II. *Cancer Treat. Rep.*, **65**, 965–972.

SAITO Y, MORI K, TOMINAGA K, YOKOI K AND MIYAZAWA N. (1990). Phase II study of 5-day continuous infusion of cis-diamminedichloroplatinum (II) in the treatment of non-small cell lung cancer. *Cancer Chemother. Pharmacol.*, **26**, 389–392.

SCHABEL FM, TRADER MW, LASTER WR, CORBETT TH AND GRISWOLD DP. (1979). Cis-dichlorodiammineplatinum (II): combination chemotherapy and cross-resistance studies with tumours of mice. *Cancer. Treat. Rep.*, **63**, 1459–1473.

SEIFERT P, BAKER LH, REED ML AND VAITKEVICIUS VK. (1975). Comparison of continuously infused 5-fluorouracil with bolus injection in treatment of patients with colorectal adenocarcinoma. *Cancer*, **36**, 123–128.

SIKIC BI. (1986). Theoretical, clinical and pharmacokinetic aspects of cancer chemotherapy administered by continuous infusion. In *Clinical Applications of Continuous Infusion Chemotherapy and Concomitant Radiation Therapy*, Rosenthal CJ and Rotman M (eds) pp.3–12. Plenum Press: New York.

VERWEIJ J, DE JONG PC, DE MULLER PHM, VAN DER BROEK P, ALEXIEVA-FIGUSCH J, VAN PUTTEN WLJ, SCHORNAGEL JH, RAVASZ LA, SNOW GB AND VERMORKEN JB. (1989). Induction chemotherapy with cisplatin and continuous infusion 5-fluorouracil in locally far-advanced head and neck cancer. *Am. J. Clin. Oncol.*, **12**, 420–424.

VOGELZANG NJ. (1984). Continuous infusion chemotherapy: a critical review. *J. Clin. Oncol.*, **2**, 1289–1304.

WEIDEN PL, EINSTEIN AB AND RUDOLPH RH. (1985). Cisplatin bolus and 5-FU infusion chemotherapy for non-small cell lung cancer. *Cancer Treat. Rep.*, **69**, 1253–1255.


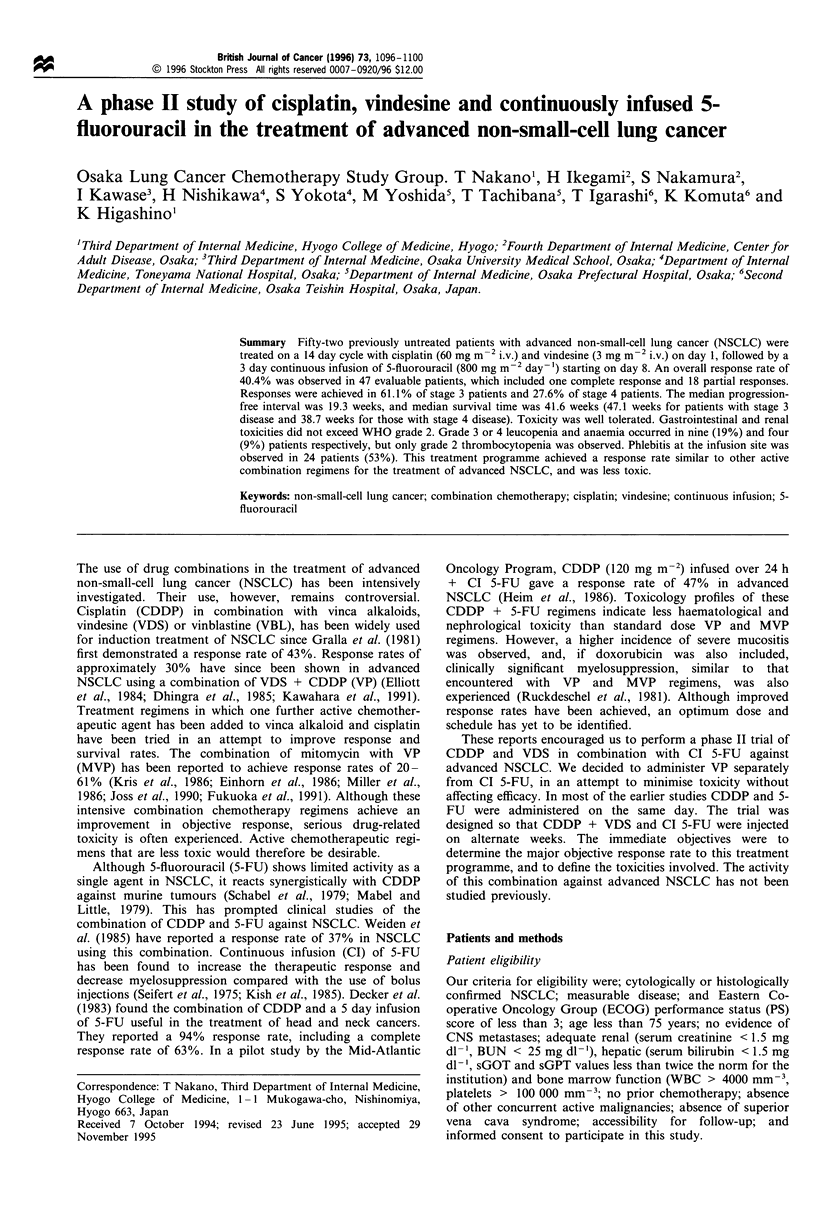

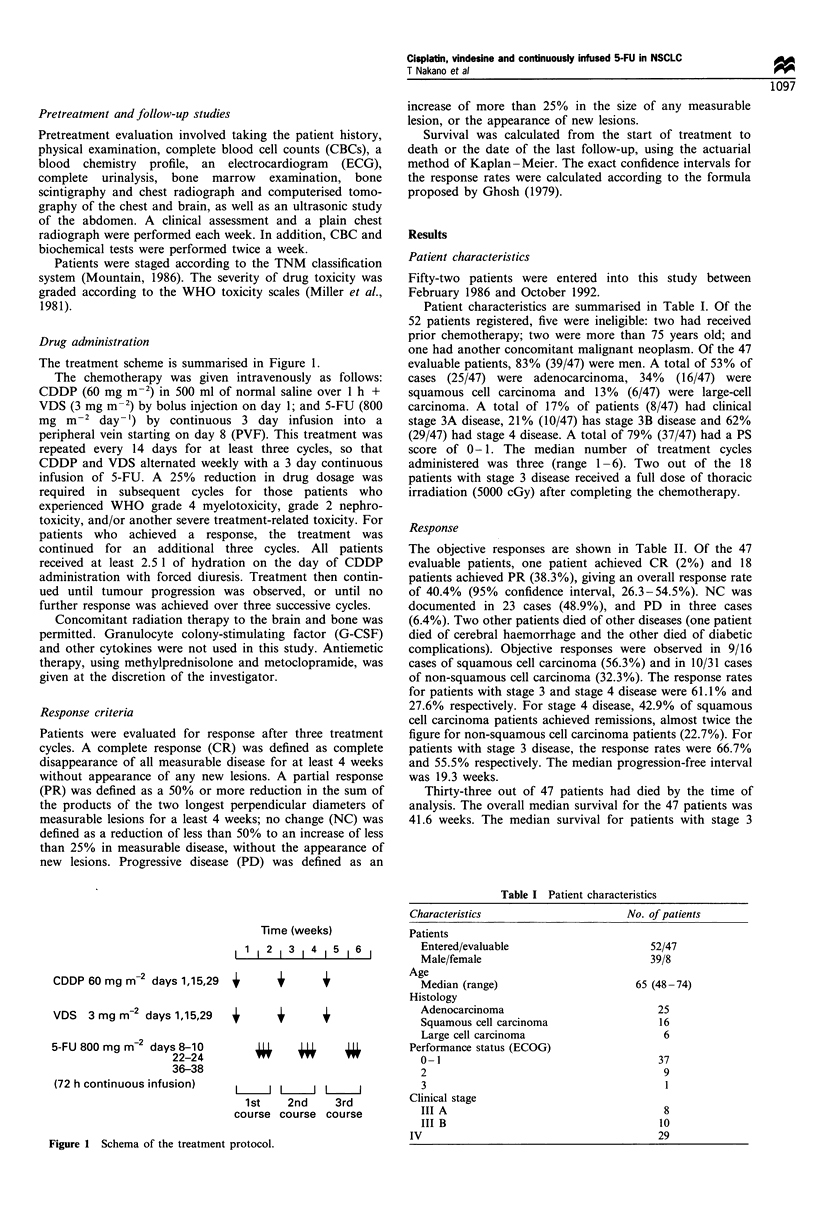

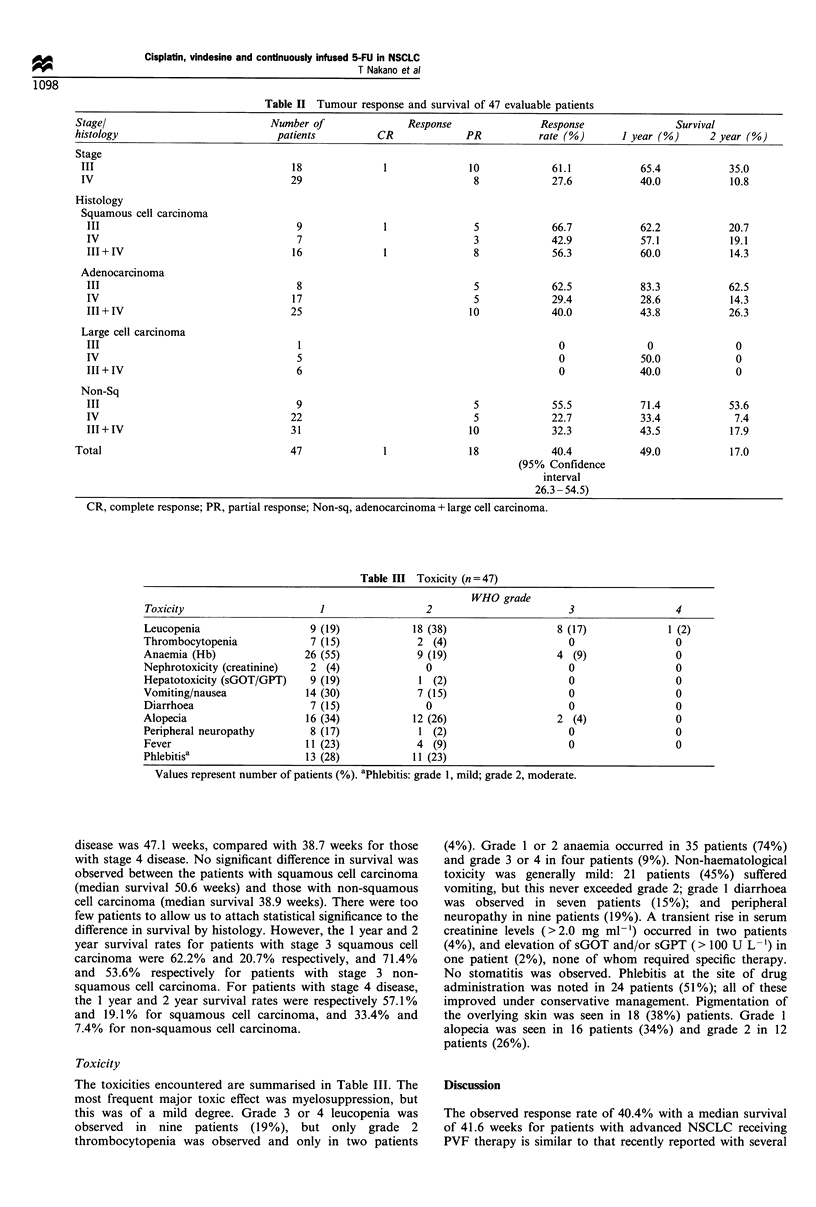

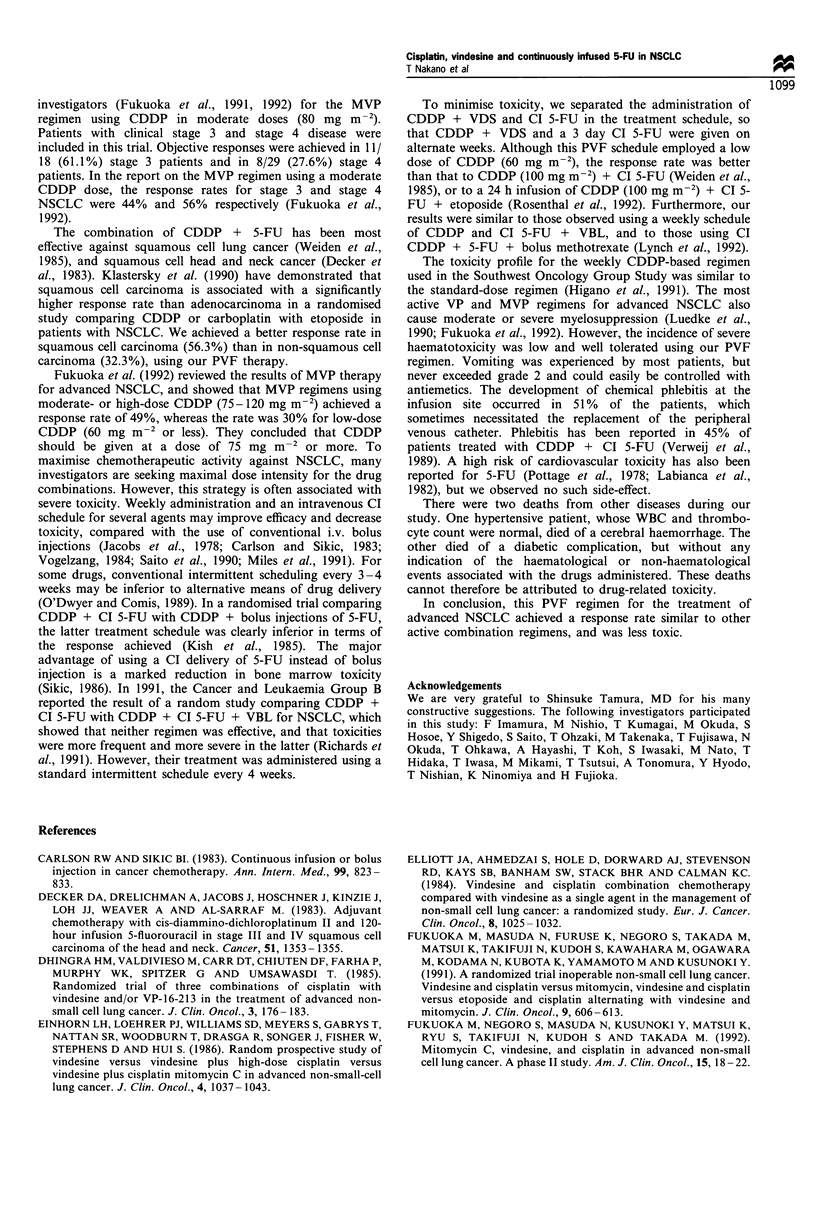

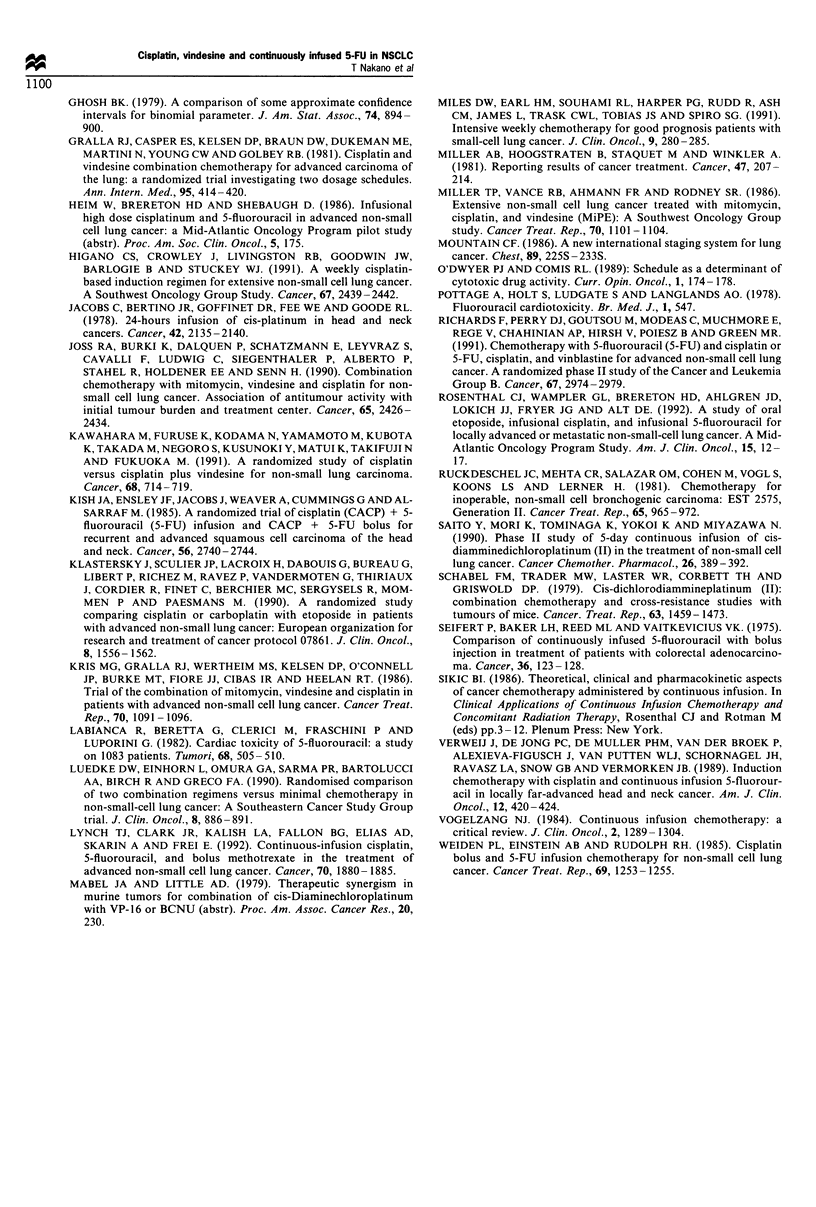


## References

[OCR_00512] Carlson R. W., Sikic B. I. (1983). Continuous infusion or bolus injection in cancer chemotherapy.. Ann Intern Med.

[OCR_00515] Decker D. A., Drelichman A., Jacobs J., Hoschner J., Kinzie J., Loh J. J., Weaver A., Al-Sarraf M. (1983). Adjuvant chemotherapy with cis-diamminodichloroplatinum II and 120-hour infusion 5-fluorouracil in Stage III and IV squamous cell carcinoma of the head and neck.. Cancer.

[OCR_00525] Dhingra H. M., Valdivieso M., Carr D. T., Chiuten D. F., Farha P., Murphy W. K., Spitzer G., Umsawasdi T. (1985). Randomized trial of three combinations of cisplatin with vindesine and/or VP-16-213 in the treatment of advanced non-small-cell lung cancer.. J Clin Oncol.

[OCR_00529] Einhorn L. H., Loehrer P. J., Williams S. D., Meyers S., Gabrys T., Nattan S. R., Woodburn R., Drasga R., Songer J., Fisher W. (1986). Random prospective study of vindesine versus vindesine plus high-dose cisplatin versus vindesine plus cisplatin plus mitomycin C in advanced non-small-cell lung cancer.. J Clin Oncol.

[OCR_00540] Elliott J. A., Ahmedzai S., Hole D., Dorward A. J., Stevenson R. D., Kaye S. B., Banham S. W., Stack B. H., Calman K. C. (1984). Vindesine and cisplatin combination chemotherapy compared with vindesine as a single agent in the management of non-small cell lung cancer: a randomized study.. Eur J Cancer Clin Oncol.

[OCR_00548] Fukuoka M., Masuda N., Furuse K., Negoro S., Takada M., Matsui K., Takifuji N., Kudoh S., Kawahara M., Ogawara M. (1991). A randomized trial in inoperable non-small-cell lung cancer: vindesine and cisplatin versus mitomycin, vindesine, and cisplatin versus etoposide and cisplatin alternating with vindesine and mitomycin.. J Clin Oncol.

[OCR_00554] Fukuoka M., Negoro S., Masuda N., Kusunoki Y., Matsui K., Ryu S., Takifuji N., Kudoh S., Takada M. (1992). Mitomycin C, vindesine, and cisplatin in advanced non-small-cell lung cancer. A phase II study.. Am J Clin Oncol.

[OCR_00573] Gralla R. J., Casper E. S., Kelsen D. P., Braun D. W., Dukeman M. E., Martini N., Young C. W., Golbey R. B. (1981). Cisplatin and vindesine combination chemotherapy for advanced carcinoma of the lung: A randomized trial investigating two dosage schedules.. Ann Intern Med.

[OCR_00589] Jacobs C., Bertino J. R., Goffinet D. R., Fee W. E., Goode R. L. (1978). 24-hour infusion of cis-platinum in head and neck cancers.. Cancer.

[OCR_00594] Joss R. A., Bürki K., Dalquen P., Schatzmann E., Leyvraz S., Cavalli F., Ludwig C., Siegenthaler P., Alberto P., Stahel R. (1990). Combination chemotherapy with mitomycin, vindesine, and cisplatin for non-small cell lung cancer. Association of antitumor activity with initial tumor burden and treatment center.. Cancer.

[OCR_00620] Klastersky J., Sculier J. P., Lacroix H., Dabouis G., Bureau G., Libert P., Richez M., Ravez P., Vandermoten G., Thiriaux J. (1990). A randomized study comparing cisplatin or carboplatin with etoposide in patients with advanced non-small-cell lung cancer: European Organization for Research and Treatment of Cancer Protocol 07861.. J Clin Oncol.

[OCR_00630] Kris M. G., Gralla R. J., Wertheim M. S., Kelsen D. P., O'Connell J. P., Burke M. T., Fiore J. J., Cibas I. R., Heelan R. T. (1986). Trial of the combination of mitomycin, vindesine, and cisplatin in patients with advanced non-small cell lung cancer.. Cancer Treat Rep.

[OCR_00642] Luedke D. W., Einhorn L., Omura G. A., Sarma P. R., Bartolucci A. A., Birch R., Greco F. A. (1990). Randomized comparison of two combination regimens versus minimal chemotherapy in nonsmall-cell lung cancer: a Southeastern Cancer Study Group Trial.. J Clin Oncol.

[OCR_00648] Lynch T. J., Clark J. R., Kalish L. A., Fallon B. G., Elias A. D., Skarin A., Frei E. (1992). Continuous-infusion cisplatin, 5-fluorouracil, and bolus methotrexate in the treatment of advanced non-small cell lung cancer.. Cancer.

[OCR_00661] Miles D. W., Earl H. M., Souhami R. L., Harper P. G., Rudd R., Ash C. M., James L., Trask C. W., Tobias J. S., Spiro S. G. (1991). Intensive weekly chemotherapy for good-prognosis patients with small-cell lung cancer.. J Clin Oncol.

[OCR_00666] Miller A. B., Hoogstraten B., Staquet M., Winkler A. (1981). Reporting results of cancer treatment.. Cancer.

[OCR_00669] Miller T. P., Vance R. B., Ahmann F. R., Rodney S. R. (1986). Extensive non-small cell lung cancer treated with mitomycin, cisplatin, and vindesine (MiPE): a Southwest Oncology Group Study.. Cancer Treat Rep.

[OCR_00677] Mountain C. F. (1986). A new international staging system for lung cancer.. Chest.

[OCR_00687] Richards F., Perry D. J., Goutsou M., Modeas C., Muchmore E., Rege V., Chahinian A. P., Hirsh V., Poiesz B., Green M. R. (1991). Chemotherapy with 5-fluorouracil (5-FU) and cisplatin or 5-FU, cisplatin, and vinblastine for advanced non-small cell lung cancer. A randomized phase II study of the cancer and leukemia group B.. Cancer.

[OCR_00695] Rosenthal C. J., Wampler G. L., Brereton H. D., Ahlgren J. D., Lokich J. J., Fryer J. G., Alt D. E. (1992). A study of oral etoposide, infusional cisplatin, and infusional 5-fluorouracil for locally advanced or metastatic non-small-cell lung cancer. A Mid-Atlantic Oncology Program study.. Am J Clin Oncol.

[OCR_00705] Ruckdeschel J. C., Mehta C. R., Salazar O. M., Cohen M., Vogl S., Koons L. S., Lerner H. (1981). Chemotherapy for inoperable, non-small cell bronchogenic carcinoma: EST 2575, generation II.. Cancer Treat Rep.

[OCR_00711] Saito Y., Mori K., Tominaga K., Yokoi K., Miyazawa N. (1990). Phase II study of 5-day continuous infusion of cis-diamminedichloroplatinum(II) in the treatment of non-small-cell lung cancer.. Cancer Chemother Pharmacol.

[OCR_00718] Schabel F. M., Trader M. W., Laster W. R., Corbett T. H., Griswold D. P. (1979). cis-Dichlorodiammineplatinum(II): combination chemotherapy and cross-resistance studies with tumors of mice.. Cancer Treat Rep.

[OCR_00723] Seifert P., Baker L. H., Reed M. L., Vaitkevicius V. K. (1975). Comparison of continuously infused 5-fluorouracil with bolus injection in treatment of patients with colorectal adenocarcinoma.. Cancer.

[OCR_00736] Verweij J., de Jong P. C., de Mulder P. H., van der Broek P., Alexieva-Figusch J., van Putten W. L., Schornagel J. H., Ravasz L. A., Snow G. B., Vermorken J. B. (1989). Induction chemotherapy with cisplatin and continuous infusion 5-fluorouracil in locally far-advanced head and neck cancer.. Am J Clin Oncol.

[OCR_00746] Weiden P. L., Einstein A. B., Rudolph R. H. (1985). Cisplatin bolus and 5-FU infusion chemotherapy for non-small cell lung cancer.. Cancer Treat Rep.

